# Penile reconstruction in a newborn following complicated circumcision: A case report

**DOI:** 10.1016/j.ijscr.2018.11.034

**Published:** 2018-11-22

**Authors:** Alessandro Innocenti, Dario Melita

**Affiliations:** Plastic and Reconstructive Microsurgery, Careggi University Hospital, Florence, Italy

Dear editors,

we really appreciated the article titled “Penile reconstruction in a newborn following complicated circumcision: A case report” by H. Al-Hazmi, in which authors described a case report of grade V iatrogenic penile amputation on a new born, reconstructed with subcutaneous corporal remnant’s release [1]. We appreciated the surgical management of this difficult case and author’s effort to clarify advantages and disadvantages of several techniques for grade V lesions.

These cases are challenging for several reasons: penis is one of the most significant features of masculinity, its role is fundamental not only for sexual intercourse, but also for social life and self-confidence. Proposed sexual reassignment, even if understandable from a surgical and medical point of view, could not be the right options since the forced acceptance of a sexual reassignment could led to psychological distress and lack of self-confidence in the future [2].

In case of immediate surgical correction, release of subcutaneous corporal remnant probably is the most appropriate solution, but we have some elements to discuss. Coverage of the penis shaft with full-thickness skin graft could not be a proper choice since its poor elasticity limiting function, erection and causing pain and lack of functionality including impotentia coeundi in the future [[3], [4], [5]]. To avoid these problems especially during the adolescence, we retain that when the scrotal tissue is not involved in any pathological condition, dartos-fascio-myo-cutaneous flap can represent a valid surgical solution for penile shaft covering. Since its subcutaneous network supports the vascular system of the scrotum, these flaps can be considered very resistant to ischemia, permitting efficiently shaft coverage even in case of large loss of tissue. Furthermore, they permit wide variability during preoperative planning both regarding dimension and shape. Moreover, the skin of the scrotum is the most similar to the skin of the shaft, including thickness and resistance during sexual intercourse.

## Conflicts of interest

Authors DO NOT HAVE financial and personal relationships with other people or organisations that could inappropriately influence (bias) their work.

## Sources of funding

Study was not funded.

## Ethical approval

This study is exempt from ethnical approval.

## Consent

There is no need for written consent since no patient’s personal information are provided.

## Author contribution

All authors contributed to the paper.

## Registration of research studies

N/A.

## Guarantor

Guarantor of the paper is the corresponding authors.

## Declarations of interest

None.
